# OPEN SCIENCE IN GERONTOLOGY: IMPLICATIONS FOR PUBLISHING

**DOI:** 10.1093/geroni/igz038.1480

**Published:** 2019-11-08

**Authors:** Derek M Isaacowitz

**Affiliations:** Northeastern University, Boston, Massachusetts, United States

## Abstract

One big push in open science is to change journal practices to encourage a more transparent and replicable scientific record. I will start by considering why these issues are important from the perspective of a journal editor. The Transparency and Openness Promotion Guidelines were developed as a modular way for journals to encourage and/or require certain practices by authors before submitting. I will describe the TOP guidelines and will recount my experience in working to bring the Journals of Gerontology: Psychological Sciences to Level 1 of the TOP guidelines. Despite the challenges involved in changing journal practices to encourage and/or mandate greater use of open science practices, these changes will likely be coming to more journals in gerontology and beyond in the coming years, making it important for authors to be aware of changes in expectations and practices.

